# A retrospective, observational, single-centre, cohort database analysis of the haemodynamic effects of low-dose spinal anaesthesia for hip fracture surgery

**DOI:** 10.1016/j.bjao.2024.100261

**Published:** 2024-02-17

**Authors:** Stuart M. White

**Affiliations:** Department of Anaesthesia, University Hospitals Sussex NHS Foundation Trust, Brighton, UK

**Keywords:** anaesthesia, cardiovascular effects, spinal, surgery, hip fracture

## Abstract

**Background:**

Careful administration of either spinal (intrathecal) or general anaesthesia probably has a greater impact on outcomes after hip fracture surgery than which method is used *per se*. Intraoperative hypotension is associated with poorer outcomes, but appears less prevalent using lower doses of spinal anaesthesia.

**Methods:**

In this observational single-centre study, intraoperative noninvasive blood pressure data were analysed from 280 patients undergoing unilateral hip fracture surgery after the administration of hyperbaric spinal bupivacaine 0.5%, 1.3 ml (0.65 mg).

**Results:**

Mean cohort mean arterial pressure (MAP) remained within 10% of baseline (spinal injection) MAP for 97/98 (99.0%) subsequent aggregated 1-min recording intervals. The prevalences of lowest MAP <70 mm Hg and <55 mm Hg were significantly lower than historical equivalents (Anaesthesia Sprint Audit of Practice 1 and 2) (52.9% and 10.4% *vs* 71.9% and 23.8%, respectively, both <0.0001). The proportions of 10 551 MAP readings <70 mm Hg and <55 mm Hg were 6.7% and 0.4%, respectively. Forty-five (16.1%) patients had relatively persistent hypotension (MAP ≤70 mm Hg for five or more intraoperative readings), and were statistically more likely to be frail (Nottingham Hip Fracture Score ≥7/10, 37.8% *vs* 19.6%, *P*=0.0109) and be taking alpha-/beta-blockers (44.4% *vs* 24.3%, *P*=0.0099) than the remaining ‘normotensive’ cohort. Surgical anaesthesia remained effective for up to 190 min, with only one patient requiring supplemental local anaesthesia during skin closure.

**Conclusions:**

Low doses of hyperbaric spinal 0.5% bupivacaine (1.3 ml, 6.5 mg) are associated with minimal reductions in blood pressure during surgery and provide adequate duration of surgical anaesthesia. Randomised comparisons of lower *vs* higher/standard doses of spinal anaesthesia are now required to confirm outcome benefits in this vulnerable patient group.

**Clinical trial registration:**

NCT05799300.

Approximately 35 000 people who fracture a hip in the UK each year are administered spinal (intrathecal) anaesthesia for surgical repair,^1^ of whom at least 30% develop intraoperative hypotension (depending on definition^2^^,^^3^), which is associated with a greater risk of death within a month of their surgery.^4^

Recent meta-analysis^5^ indicates that the mode of anaesthesia (i.e. ‘spinal’ or ‘general’) is probably of much less importance than how carefully that anaesthesia is administered (i.e. in a standardised manner, aiming to remobilise, re-enable, and rehabilitate people shortly after surgery^6^). This supports existing national^7^ and international^8^ guidelines that recommend using lower volumes of local anaesthetic for spinal anaesthesia (e.g. bupivacaine 0.5%, 1.5 ml) than are commonly used (bupivacaine 0.5%, 2.5 ml^4^) to reduce the likelihood of perioperative hypotension. However, those recommendations were based on historical research selectively involving younger, fitter people having hip fracture surgery,^9^, ^10^, ^11^ in whom blood pressure was recorded neither particularly accurately nor often enough.

Recently, a non-proprietary method for transferring vital signs data electronically from anaesthetic monitors to storage computers has been described.^12^ Although intended for medicolegal purposes (e.g. Coronial investigations), analysis of these data could also enable accurate description of how blood pressure changes around the time of surgery, and among subsets of hip fracture patients who are normally excluded from prospective research (e.g. the very old, the very frail, people with dementia^13^). Furthermore, by comparing these data with published national data (e.g. Anaesthesia Sprint Audit of Practice, ASAP, 1^3^ and 2^4^), it would become possible to determine whether lower dose spinal anaesthesia is associated with a significantly lower prevalence of hypotension during hip fracture surgery.

The primary objective of this research, therefore, is to describe the relative changes in noninvasive systolic, mean, and diastolic arterial blood pressures collected prospectively at regular 2-min intervals from a cohort of consecutive patients undergoing hip fracture repair surgery who were administered low-dose spinal anaesthesia (hyperbaric bupivacaine 0.5%, 1.3 ml without additional opioid).

The secondary objectives of this research are (1) to analyse the significance of any differences in the prevalence of intraoperative hypotension (variably defined) using low-dose spinal anaesthesia for hip fracture repair compared with historical national ‘all-dose’ and ‘mode-dose’ (i.e. hyperbaric bupivacaine 0.5%, 2.5 ml) spinal anaesthesia data from England and Wales,^3^^,^^4^ (2) to describe the proportion of blood pressure recordings that fell below variably defined thresholds for hypotension, and (3) to determine the effective duration of surgical anaesthesia after low-dose spinal administration.

## Methods

### Trial design and participants

This retrospective, observational, single-centre, cohort database analysis was approved after proportionate ethical review by East Midlands - Derby Research Ethics Committee (IRAS 279652) on 15 June 2022. The study is registered on ClinicalTrials.gov (NCT05799300). Individual consent was not sought from study subjects.

### Trial participants

All people undergoing unilateral surgical hip fracture repair (cemented/uncemented hemiarthroplasty, dynamic hip screw, cortical screws, proximal femoral nail) administered anaesthesia by the author at Princess Royal Hospital, Hayward's Heath, UK, for whom contemporaneous vital sign (noninvasive systolic/diastolic/mean arterial blood pressure, heart rate, oxygen saturation, ventilatory frequency) data were recorded, were eligible for inclusion in this analysis. Data were available from 10 March 2017 and analysed up to 13 January 2020 (i.e. when hospital services were reconfigured in light of COVID-19).

Vital sign data were captured from anaesthetic monitors (AS3, GE Healthcare, Hatfield, UK) using hardwire connection to a double password-protected laptop computer, and transferred electronically (via NHS email) to double password-protected NHS desktop computers, before deletion from the laptop. These records are normally held for medicolegal purposes/Coronial investigation.^12^

Pseudo-anonymisation (hospital number, date of surgery) was used to link these data with patient physical and injury characteristics (sex, age, American Society of Anesthethesiologists [ASA] physiological status score, Nottingham Hip Fracture Score [NHFS^14^], Jiang risk score^14^), abbreviated mental test score (AMTS), pre-fracture level of care/mobility/comorbidity (hypertension/valvular heart disease/diabetes)/medications (alpha-with or without beta-adrenoceptor blockade/other antihypertensive/anticoagulation), process (timings, anaesthesia and surgical interventions), and outcome (died during admission/within 30 days of surgery, length of postoperative stay [LOPS]) data held on the Brighton Hip Fracture database,^15^ before data were fully anonymised for analysis. Data were held and analysed in compliance with the requirements of the General Medical Council's Good Clinical Practice, the Data Protection Act 2018, and the European Union General Data Protection Regulations 2016/679.

All study participants received broadly consistent institutional and anaesthetic interventions. During their hospital admission, patients followed a standardised, multidisciplinary hip fracture care pathway, co-ordinated by senior orthogeriatricians. Hip fracture patients requiring surgery (>99.8%) were identified and discussed at a pre-theatre trauma meeting, having usually been admitted to hospital within the previous 24 h (longer if further management was required, particularly of their anticoagulation).

### Anaesthetic techniques

After transfer from the hip fracture unit direct to the anaesthetic room, patients were checked in according to institutional and World Health Organization (WHO) criteria. With venous access having been routinely established on the hip fracture unit, patients were commenced on 1 L i.v. crystalloid and connected to routine noninvasive electrocardiogram, pulse oximetry and blood pressure monitoring, the latter set to record every 2 min. After correct side confirmation, a supra-inguinal fascia iliaca block was administered (using an antiseptic technique, a linear ultrasound probe, and infiltration of plain bupivacaine 0.375%, 20 ml + lignocaine 0.5%, 20 ml via an in-plane 50 mm 30° facet bevel stimulating single-shot nerve block needle (Stimuplex®, B Braun, Sheffield, UK), unless the patient had received a fascia iliaca block within the previous 4 h. After commencement of 2–3 L min^−1^ oxygen via a CO_2_ sampling-enabled facemask (adult Sentri^TM^ EcoLite^TM^, Intersurgical, Wokingham, UK), 20–40 mg (30–60 mg in younger, fitter patients) propofol sedation was administered before positioning the patient comfortably in the lateral decubitus position with the operative side dependant. Using a strictly aseptic technique, lignocaine 1%, 2–3 ml was infiltrated subcutaneously and into the interspinous space via a 23 G hypodermic needle, before accessing the (estimated) L2–3 intrathecal space via a 25 G pencil-point spinal needle (Sprotte NRFit®, Pajunk UK Medical Products Ltd., Newcastle upon Tyne, UK), confirming cerebrospinal fluid flow and administering hyperbaric bupivacaine 0.5%, 1.3 ml by low-pressure injection over 30 s to 1 min. Having confirmed a satisfactory blood pressure 2–3 min after spinal injection, patients were transferred to the operating theatre, where the effectiveness of their block was confirmed: during transfer from bed to operating table, during surgical skin preparation (abduction of the hip for hemiarthroplasty, distraction of the fracture for internal/external fixation) and by pre-incision sharp surgical stimulus. Boluses of propofol sedation (20–40 mg) were administered i.v. according to patient agitation. Boluses of vasopressors (metaraminol 0.5 mg, ephedrine 6 mg) were administered to maintain a subjectively ‘suitable’ mean arterial pressure (MAP) and heart rate. Hemiarthroplasties were performed in a lateral decubitus position, and fixation in a supine position, by senior (consultant/supervised specialist trainees) surgeons. I.V. antibiotic prophylaxis (teicoplanin 400–600 mg + gentamicin 160 mg) and tranexamic acid (1 g) were administered before incision. Red blood cell salvage was not used routinely. After surgery, patients were disconnected from i.v. fluids and oxygen (unless indicated clinically) and admitted to the post-anaesthesia care unit, sitting up at 45–60° in their ward beds, before discharge back to the orthogeriatric hip fracture unit care.

Data from the ASAP 2 study were collected and handled as described previously.^3^^,^^4^

## Outcomes

The primary outcome measure was mean (standard deviation, sd) systolic, mean, and diastolic noninvasive blood pressures (NIBPs) taken at 2-min intervals from all patients included in the study.

The secondary outcome measures were (1) the prevalence of hypotension, variably defined as (relative) decrease from baseline (t_0_ time point of spinal anaesthesia infiltration) of: systolic blood pressures (SBPs) >20%/30%; MAPs >20%/30%; and (absolute) lowest: SBPs <90/100 mm Hg and MAPs <70/55 mm Hg; (2) the proportions of all SBP/MAP blood pressure readings that fell below these definition thresholds; (3) quantification of the effective duration in minutes of low-dose spinal anaesthesia.

### Sample size calculation

The sample size was determined pragmatically. Previously, the largest prospective trials comparing hypotension after different doses of spinal anaesthesia have reported NIBP recording intervals of 5 min in 10–37 patients.^9^, ^10^, ^11^ From historical Brighton Hip Fracture Database data (for the author), patients spend ∼20 min in the anaesthetic room, then 15 min being prepared for surgery and 60 min undergoing surgery. In order to calculate a stable mean (sd) for the primary outcome measure, the study aimed to record NIBP data from at least 37 patients for every 2-min recording cycle during their estimated (20+15+60=) 95 min stay in the operating theatres, generating ∼4162 data points in total (i.e. three systolic/mean arterial/diastolic blood pressures × 37 patients × 37.5 recordings per patient), enabling sufficient descriptive graphical representation of the primary outcome measure (i.e. blood pressure *vs* time).

### Statistical analysis

Normally distributed data (timings, vasopressor doses administered) are described by their mean and sd, and compared using unpaired *t*-tests. Skewed or non-continuous data (age, NHFS, AMTS, LOPS) are described by their median (inter-quartile range, IQR [range]), and compared using two-tailed Fisher's exact tests. Binary logistic regression calculations were modelled in Excel, using Solver (both Microsoft 2011, Redmond, WA, USA).

## Results

### Trial population

**Fig 1:**
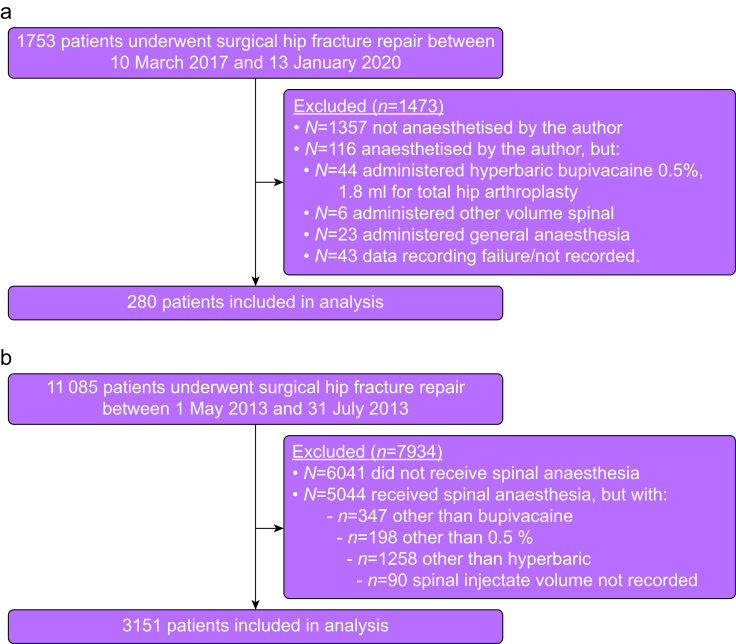
Flow chart of patients included in the analysis (a) contemporary data (b) historical data from the ASAP 2 study.^4^.

Between 10 March 2017 and 13 January 2020, 1753 patients underwent surgery to repair their hip fracture. The author administered anaesthesia to 396 (22.6%) of these people, 280 (70.7%) of whom met the criteria for inclusion in this retrospective analysis (Fig 1a): patient data are shown in Table 1. Data recording generated 31 653 blood pressure data points in total (i.e. three systolic/mean/diastolic blood pressures × 280 patients × mean (sd) 38 (10) recordings per patient). Historical, comparative ASAP 2 inclusion data for 3151 patients are shown in Fig 1b.

**Table 1:** Patient characteristics, perioperative and outcome data for the 280 people with hip fracture included in the analysis. Cemented hemiarthroplasty = Exeter prosthesis; uncemented hemiarthroplasty = Austin-Moore prosthesis. AMTS, Abbreviated Mental Test Score (out of 10); ASA, American Society of Anesthesiologists physiological status; IQR, inter-quartile range; NHFS, Nottingham Hip Fracture Score (out of 10)^14^ Jiang,^14^; sd, standard deviation. ^∗^One patient required local anaesthesia infiltration to the cephalad end of their surgical wound during closure.

Patient characteristic	*N* (%)	Mean (sd)	Median (IQR [range])
Age (yr)			87 (81–91 [52–101])
Sex Female	205 (73.2)		
Male	75 (16.8)		
ASA 1	4		
2	54		
3	187		
4	35		
NHFS			5 (4–6 [0–9])
0–6	217		
7–10	63		
Jiang			30 (16.75–43.25 [0–94])
AMTS			8 (4–10 [0–10])
0–6	178 (63.6)		
7–10	102 (36.4)		
**Pre-fracture care**			
*Self care*	206 (73.6)		
Unsupported	144 (51.4)		
Home help	45 (16.1)		
Sheltered	17 (6.1)		
*Receiving care*	74 (26.4)		
Residential	42 (19.0)		
Nursing/inpatient	32 (11.4)		
**Pre-fracture mobility**			
*Unsupported*	100 (35.7)		
*Supported*	180 (64.3)		
Stick/2	94 (33.6)		
Frame	83 (29.6)		
Chair/bedbound	3 (1.1)		
**Pre-fracture comorbidity**			
*Diabetes*	39 (13.9)		
*Hypertension*	152 (54.3)		
*Valvular heart disease*	59 (21.1)		
Ejection systolic murmur present	41 (14.6)		
Echocardiograph available for review	8 (19.5)		
*Medications*			
(Alpha-) Beta-blockade	77 (27.5)		
Other antihypertensive	122 (43.6)		
Anticoagulation	126 (45.0)		
Apixaban	14 (5.0)		
Aspirin	50 (17.9)		
Clopidogrel	20 (7.1)		
Rivaroxaban	19 (6.8)		
Warfarin	12 (4.3)		
Combination/other	11 (3.9)		
**Perioperative data**			
**Timings (min)**			
Preoperative wait		1271 (609)	
Anaesthetic time		24 (7)	
Preparation time		15 (5)	
Surgical time		50 (17)	
Theatre time		67 (22)	66 (51–80 [19–190])
**Anaesthesia**			
Fascia iliaca block coadministered	278 (99.3)		
Local anaesthesia infiltration	2 (0.7)^∗^		
Propofol sedation coadministered	278 (99.3)		
Metaraminol administered (mg)	104 (37.1)	0.9 (0.7)	0.75 (0.5–1 [0.5–6])
Ephedrine administered (mg)	79 (28.2)	6.5 (1.6)	6 (6–6 [3–15])
Intra-arterial blood pressure monitoring	0		
1 L I.V. crystalloid administered	266 (95.0)		
Estimated blood loss >500 ml	0		
Suspected bone cement reaction	0		
**Surgery fracture type/implant**			
*Intracapsular*	143 (51.1)		
Cemented hemiarthroplasty	116 (41.4)		
Uncemented hemiarthroplasty	2 (0.7)		
Dynamic cortical screws	(24 (8.6)		
*Extracapsular*			
Dynamic hip screw	37 (13.2)		
Proximal femoral nail	100 (35.7)		
**Outcomes**			
Died during inpatient admission	13 (4.6)		
Died within 30 days after surgery	15 (5.4)		
Length of postoperative stay (days)			14 (10–20 [2–83])

## Primary outcome

**Fig 2:**
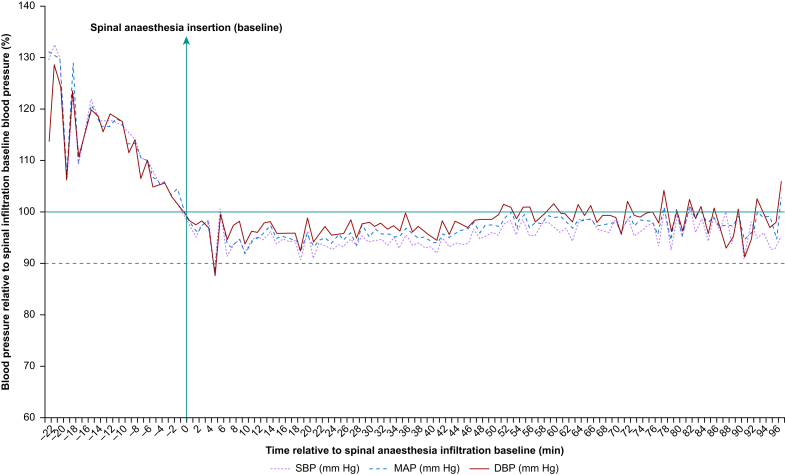
Mean relative decreases in systolic, mean, and diastolic blood pressures over time compared with baseline (spinal anaesthesia infiltration at t0) for 280 people administered hyperbaric bupivacaine 0.5%, ≤1.3 ml for unilateral hip fracture repair (excluding total hip arthroplasty). For clarity, data points averaged from fewer than 10 patients are excluded (Supplementary Table S1). DBP, diastolic blood pressure; MAP, mean arterial pressure; SBP, systolic blood pressure.

The mean relative decreases in systolic, mean, and diastolic blood pressures over time compared with baseline (spinal anaesthesia infiltration at t_0_ [i.e. the primary objective]) are shown graphically in Fig 2 (numerical mean [sd] are provided in Supplementary Table S1).

## Secondary outcomes

The prevalence of the lowest blood pressure values recorded that fell below various definition thresholds for ‘hypotension’ among the 280 sets of patient data analysed (and compared with national data from ASAP) are shown in Table 2.

**Table 2:** Prevalence (%) of patients with lowest blood pressure below defined ‘hypotensive’ threshold, by volume of hyperbaric bupivacaine 0.5% spinal anaesthesia administered. Compared with data from the 280 study patients, ‘≤1.3 ml’ from the ASAP data defines an equivalent volume, ‘≤1.5 ml’ the volume above which SBP fell by more than the ‘least worst’ definition of hypotension (i.e. a relative decrease >20%) and 2.5 ml the mode volume administered in England and Wales. *P*-values indicate statistical significance differences between ASAP cohorts compared with current study cohort. ASAP, Anaesthesia Sprint Audit of Practice; MAP, mean arterial pressure; SBP, systolic blood pressure.

Definition	Current 1.3 ml *n*=280	ASAP 1 all, *n*=3151	*p*	ASAP <1.3 ml, *n*=44	*p*	ASAP ≤1.5 ml, *n*=121	*p*	ASAP 2.5 ml, *n*=471	*p*
Relative hypotension									
Decrease in SBP >20%	65.7	86.2	<0.0001	61.4	0.6112	70.2	0.4184	88.1	<0.0001
Decrease in SBP >30%	41.8	64.5	<0.0001	29.5	0.1386	39.7	0.7406	67.9	<0.0001
Decrease in MAP >20%	62.9	85.8	<0.0001	54.5	0.3189	68.6	0.3064	87.7	<0.0001
Decrease in MAP >30%	40.4	62.9	<0.0001	22.7	0.0294	40.5	1.0000	66.6	<0.0001
Absolute hypotension									
SBP <100 mm Hg	61.8	67.4	0.0548	31.8	0.0003	46.3	0.0043	71.1	0.0098
SBP <90 mm Hg	36.4	44.5	0.0099	18.2	0.0169	27.3	0.0845	49.0	0.0008
MAP <70 mm Hg	52.9	71.9	<0.0001	38.6	0.1042	57.0	0.4475	74.9	<0.0001
MAP <55 mm Hg	10.4	23.8	<0.0001	4.5	0.2815	9.1	0.8561	27.6	<0.0001

The proportion of blood pressure readings recorded that fell below various definition thresholds for ‘hypotension’ among the patients' data analysed are shown in Table 3.

**Table 3:** Proportion of 10 551 blood pressure readings below various definition thresholds for ‘hypotension’. MAP, mean arterial pressure; SBP, systolic blood pressure.

Definition	*N* (%)
Relative	
Decrease in SBP >20%	1966 (18.6)
Decrease in SBP >30%	825 (7.8)
Decrease in MAP >20%	2014 (19.9)
Decrease in MAP >30%	831 (7.9)
Absolute	
Lowest SBP <100 mm Hg	1123 (10.6)
Lowest SBP <90 mm Hg	415 (3.9)
Lowest MAP <70 mm Hg	712 (6.7)
Lowest MAP <55 mm Hg	42 (0.4)

The median (IQR [range]) duration of theatre time and outcomes are shown in Table 1. One patient required additional local anaesthesia infiltration to the superior margin of their surgical wound during closure.

During data analysis, it became apparent that a subgroup of the patient cohort had recorded several low intraoperative MAPs. Arbitrarily defining patients in this group by having MAP ≤70 mm Hg for five or more readings (equivalent to 10 min duration of hypotension), the prevalence of potential causative factors in this group compared with the remainder of the cohort are shown in Table 4. In the low-pressure subgroup, five of the six patients who died during admission (and four of the five patients who died within 30 days of surgery) were ‘frail’ and adrenoceptor-blocked before operation.

**Table 4:** Comparison of potentially causative factors, vasopressor administration, and outcomes between hip fracture patient ‘low-pressure’ subgroup (i.e. MAP ≤70 mm Hg for five or more readings), and ‘normal-pressure’ remainder of the cohort. Age ≥86 yr and NHFS ≥7/10 (as a surrogate marker for frailty) have been previously identified as risk factors for increased perioperative mortality/institutionalisation after hip fracture.^16^ ‘Likely aortic stenosis’ identifies patients with a clinical/echocardiographic diagnosis of aortic stenosis with or without an audible ejection systolic murmur before operation during cardiac auscultation. A delay of LOPS ≥36 h represents potentially two episodes of preoperative starvation with or without fluid replacement. LOPS length of (in)patient stay; MAP mean arterial pressure; NHFS, Nottingham Hip Fracture Score; sd, standard deviation.

Parameter	‘Low-pressure’, *N*=45	‘Normal-pressure’, *N*=235	*P*-value	Regression coefficient
Alpha- with or without beta-blockade, *N* (%)	20 (44.4)	57 (24.3)	0.0099	0.88
Other antihypertensive, *N* (%)	23 (51.1)	99 (42.1)	0.3250	0.46
Diabetes, *N* (%)	7 (15.6)	32 (13.6)	0.8139	−0.04
Age ≥86 yr, *N* (%)	30 (66.7)	137 (58.3)	0.3236	0.17
‘Frail’ NHFS ≥7/10, *N* (%)	17 (37.8)	46 (19.6)	0.0109	0.78
Hypertension, *N* (%)	26 (57.8)	126 (53.6)	0.6283	−0.29
Likely aortic stenosis, *N* (%)	7 (15.6)	34 (14.5)	0.8200	−0.06
Preoperative delay ≥36 h, *N* (%)	3 (6.7)	27 (11.5)	0.4374	−0.72
Mean (sd) metaraminol dose (mg)	0.7 (0.5)	0.3 (0.7)	0.0003	
Mean (sd) ephedrine dose (mg)	2.3 (1.0)	1.7 (1.7)	0.0227	
Died during admission, *N* (%)	6 (13.3)	7 (3.0)	0.0089	
Died within 30 days of surgery, *N* (%)	5 (11.1)	10 (4.3)	0.0734	
Mean (sd) LOPS (days)	17 (8)	17 (11)	0.4567	

## Discussion

In this observational database study, anaesthesia involving low-dose spinal hyperbaric bupivacaine 0.5% (1.3 ml [=6.5 mg], without additional opioid) was associated with relative normotension. Mean cohort MAP remained within 10% of baseline MAP for 97 of the 98 (99.0%) aggregated 1-min recording time intervals (Fig 1, Supplementary Table S1). The nadir mean cohort MAP reading occurred ∼5 min after spinal administration, which anaesthetists should account for when transferring patients between the anaesthetic room and operating theatre. Although nadir blood pressures occurred relatively abruptly, they approximated to only a 10% relative decline from baseline at spinal anaesthesia administration, and normotension was restored equally abruptly, with only a third of patients requiring (low dose) vasopressor administration (median metaraminol 0.75 mg, ephedrine 6 mg).

Within the mean cohort data, there was considerable variation in how much the blood pressure changed in individual patients (relative median change MAP 21% (IQR 20–23% [range 7–36%], Supplementary Table S1). Proportionately, however, relatively few of the systolic and mean blood pressures recorded fell below thresholds that have previously been associated with increased mortality and which have occurred with comparably greater prevalence in both hip fracture^3^^,^^4^ and general surgical populations^17^, ^18^, ^19^ ([prevalence] lowest MAP <70 mm Hg 52.9% and <55 mm Hg 10.4% *vs* 71.9% and 23.8% [ASAP 1^3^], respectively [Table 2]; [proportion] MAPs <70 mm Hg 6.7% and <55 mm Hg 0.4% [Table 3]).

Of note, greater mean relative decreases in SBP and MAP (∼30%) were observed *before* spinal anaesthesia administration, confirming earlier observational data that challenge the definition of a reference ‘baseline’ on which subsequent anaesthetic interventions might be administered,^18^, ^19^, ^20^ and indicating important potential hypotensive effects related to both pain (during ward/theatre transfer)/analgesia (nerve block) and anxiolysis/sedation with propofol (for lateral positioning).

Interestingly, the 45 hip fracture patients with relatively persistent hypotension (i.e. MAP ≤70 mm Hg for five or more readings) were statistically more likely to be frail (i.e. have an NHFS ≥7/10), be taking alpha-/beta-blockers, experience longer preoperative delays, and to die during hospital admission than the ‘normotensive’ remainder of the study cohort (Table 4), indicating future avenues for research in identifying potentially ‘endotypical’ patients at mortal/morbid risk of intraoperative hypotension, and, again, alerting anaesthetists to perhaps more intensive management of such patients.^21^^,^^22^

In contrast to the few randomised controlled trials (RCTs) reporting hypotensive events after variable doses of spinal bupivacaine,^9^, ^10^, ^11^^,^^23^ a comparative strength of this study concerns its involvement of a properly representative hip fracture population. It might be expected that including data on older, frailer, co-morbid patients might be associated with more prevalent hypotensive episodes, greater absolute falls, and wider variations in blood pressure, related to impaired homeostasis. However, the data reported in this observational study are quantitatively similar to those reported in previous RCTs^9^, ^10^, ^11^ and observational studies,^3^^,^^4^ indicating similarly prevalent absolute and relative hypotensive episodes (Table 2) and magnitude of pressure falls after low-dose spinal administration (∼10% *vs* 15^9^, ^10^, ^11^–20%^10^), that statistically are significantly smaller than those occurring after ‘normal’ dose spinal anaesthesia administration (i.e. hyperbaric bupivacaine 0.5%, 2.5 ml [12.5 mg]) (Table 2).^3^^,^^4^^,^^23^

Indeed, those quantitative similarities between the data presented and historical data reported for low-dose spinal anaesthesia should reassure readers that the confounding variables normally associated more frequently with observational, compared with RCT, data are unlikely to be influencing the results presented here to a significant degree that might alter the conclusions drawn from those data. The main weakness of this study is that potentially vasoactive stimuli may have influenced blood pressure changes other than those attributable to spinal bupivacaine administration, including analgesia (immobility, preoperative nerve block, fracture type^24^); other stimulation (movement, surgical percussion, hip relocation); bone cement implantation^25^; active warming; sedation (propofol dose/frequency/total dose); and vasopressor use (alpha-/beta-blocker dose/timing/frequency/total dose). However, previous RCTs also did not take such confounding variables into account, excepting the use of objective vasopressor administration below predefined blood pressure thresholds (SBP <90 mm Hg^9^–100 mm Hg,^11^ or a decrease of SBP >20%^10^/MAP >25% from baseline^9^).

Despite the lower dose of spinal bupivacaine used, surgical anaesthesia remained effective for up to 190 min, with only one of 280 patients requiring supplemental local anaesthesia to the upper margin of their surgical skin incision during closure. These data should allay understandable concerns about low-dose spinal bupivacaine methods providing sufficient duration of surgical anaesthesia,^26^^,^^27^particularly when co-administered with an ultrasound-guided fascia iliaca block.

Similarly, these data should re-assure clinicians that using low-dose spinal anaesthesia does not seem to be associated with adverse patient outcomes. Indeed, both the observed incidence of 30-day postoperative mortality and the observed median LOPS compared favourably with time-equivalent national figures (5.4% *vs* 6.6%, 15.0 *vs* 15.5 days, respectively).

In conclusion, the observational data presented here suggest that low doses of hyperbaric spinal bupivacaine (0.5%, 1.3 ml, 6.5 mg) are associated with minimal reductions in blood pressure during surgery in a representative sample of hip fracture patients, compared with historical data. These data validate those from RCTs which support the preferential use of lower doses of hyperbaric spinal bupivacaine than are currently used (typically 0.5%, 2.5 ml, 12.5 mg), with the aim of reducing both the prevalence and duration of hypotensive episodes intraoperatively (and so, potentially, reducing poor mortal and morbid outcomes). Low-dose spinal bupivacaine provided adequate surgical anaesthesia in this study.

The mode of anaesthesia probably has little influence on commonly measured outcomes after hip fracture surgery.^5^, ^6^, ^7^, ^8^ Instead, researching the improved delivery of both spinal and general anaesthesia within intraoperative care would seem more likely to improve patients' recovery trajectories. In the first instance, this will require future research focus being redirected away from general *vs* regional anaesthesia dualism towards comparisons of appropriate outcomes (hypotension, pain, confusion, mobility) after lower *vs* higher dose anaesthesia.

## Declaration of interests

SW convened the International Fragility Fractures Network Consensus Group on the principles of anaesthesia for patients with hip fracture. SW was a member of the Association of Anaesthetists Working Party on the management of hip fractures, and is a scientific advisor to the World Hip Trauma Evaluation (WHiTE). No other interests and no external funding declared.

## Data availability

Study data are currently on password-protected databases at University Hospitals Sussex NHS Foundation Trust, but are freely available for access upon application to the author.
