# Supplementary material for: A retrospective, observational, single-centre, cohort database analysis of the haemodynamic effects of low-dose spinal anaesthesia for hip fracture surgery
Source: BJA Open. 2024 Feb 17;9:100261. doi: 10.1016/j.bjao.2024.100261 (PMC10882127; doi:10.1016/j.bjao.2024.100261)
Supplement: Multimedia component 1 [file mmc1.doc]

| Time relative to spinal anaesthesia infiltration, ± mins | Blood pressure relative to spinal anaesthesia infiltration, mean SD | | | Number  of  patients, N |
| --- | --- | --- | --- | --- |
|  | Systolic | Diastolic | Mean |  |
| -41 | 115 | 114 | 129 | 1 |
| -40 | 118 | 133 | 130 | 1 |
| -39 |  |  |  | 0 |
| -38 | 101 | 82 | 103 | 1 |
| -37 |  |  |  | 0 |
| -36 |  |  |  | 0 |
| -35 | 101 (23) | 111 (21) | 114 (10) | 4 |
| -34 | 98 | 97 | 112 | 1 |
| -33 | 103 (22) | 128 (25) | 117 (13) | 4 |
| -32 | 80 (3) | 89 (20) | 93 (11) | 2 |
| -31 | 110 (17) | 122 (23) | 114 (12) | 5 |
| -30 | 102 (27) | 98 (7) | 99 (12) | 3 |
| -29 | 102 (26) | 113 (39) | 111 (29) | 6 |
| -28 | 110 (36) | 113 (33) | 114 (26) | 4 |
| -27 | 113 (20) | 95 (21) | 107 (13) | 4 |
| -26 | 127 (37) | 118 (35) | 131 (41) | 7 |
| -25 | 118 (31) | 120 (13) | 133 (42) | 4 |
| -24 | 136 (23) | 122 (32) | 128 (29) | 7 |
| -23 | 104 (39) | 104 (26) | 118 (46) | 5 |
| -22 | 129 (25) | 114 (27) | 131 (36) | 12 |
| -21 | 132 (34) | 129 (25) | 130 (27) | 16 |
| -20 | 130 (28) | 124 (27) | 129 (32) | 19 |
| -19 | 108 (23) | 106 (22) | 108 (18) | 18 |
| -18 | 128 (30) | 124 (32) | 129 (30) | 27 |
| -17 | 111 (16) | 111 (17) | 109 (19) | 26 |
| -16 | 117 (20) | 116 (27) | 117 (22) | 47 |
| -15 | 122 (25) | 120 (28) | 121 (23) | 40 |
| -14 | 118 (24) | 119 (28) | 118 (24) | 78 |
| -13 | 117 (25) | 116 (27) | 117 (25) | 62 |
| -12 | 118 (21) | 119 (23) | 117 (20) | 134 |
| -11 | 117 (18) | 118 (22) | 118 (18) | 76 |
| -10 | 117 (20) | 118 (22) | 117 (21) | 159 |
| -9 | 115 (20) | 111 (24) | 113 (24) | 76 |
| -8 | 114 (21) | 114 (22) | 114 (22) | 162 |
| -7 | 111 (19) | 107 (20) | 106 (18) | 61 |
| -6 | 110 (20) | 110 (21) | 110 (21) | 159 |
| -5 | 108 (22) | 105 (22) | 107 (19) | 78 |
| -4 | 105 (13) | 105 (15) | 106 (15) | 158 |
| -3 | 106 (17) | 106 (15) | 105 (17) | 49 |
| -2 | 103 (8) | 103 (11) | 104 (12) | 179 |
| -1 | 102 (10) | 102 (12) | 105 (15) | 37 |
| 0 | 100 (0) | 100 (0) | 100 (0) | 280 |
| 1 | 97 (5) | 98 (9) | 98 (7) | 31 |
| 2 | 95 (9) | 98 (11) | 96 (11) | 166 |
| 3 | 98 (14) | 98 (14) | 97 (13) | 51 |
| 4 | 99 (17) | 97 (18) | 98 (17) | 97 |
| 5 | 88 (13) | 87 (15) | 89 (12) | 28 |
| 6 | 99 (19) | 100 (24) | 101 (21) | 37 |
| 7 | 92 (25) | 95 (26) | 94 (25) | 42 |
| 8 | 94 (25) | 97 (24) | 94 (24) | 53 |
| 9 | 95 (25) | 98 (29) | 95 (25) | 75 |
| 10 | 92 (19) | 94 (22) | 92 (18) | 81 |
| 11 | 94 (28) | 96 (27) | 94 (25) | 114 |
| 12 | 95 (25) | 96 (27) | 95 (24) | 114 |
| 13 | 95 (23) | 98 (26) | 96 (24) | 117 |
| 14 | 96 (22) | 98 (26) | 97 (23) | 125 |
| 15 | 94 (23) | 96 (27) | 95 (24) | 114 |
| 16 | 95 (21) | 96 (27) | 95 (22) | 139 |
| 17 | 94 (23) | 96 (23) | 95 (21) | 115 |
| 18 | 95 (21) | 96 (26) | 95 (22) | 138 |
| 19 | 91 (22) | 92 (26) | 93 (23) | 115 |
| 20 | 97 (21) | 99 (26) | 96 (21) | 140 |
| 21 | 91 (19) | 94 (23) | 93 (21) | 127 |
| 22 | 94 (21) | 96 (24) | 94 (20) | 137 |
| 23 | 93 (18) | 97 (21) | 95 (18) | 126 |
| 24 | 93 (20) | 95 (23) | 94 (19) | 138 |
| 25 | 94 (19) | 96 (22) | 96 (20) | 127 |
| 26 | 93 (20) | 96 (24) | 94 (20) | 145 |
| 27 | 95 (20) | 99 (26) | 96 (21) | 117 |
| 28 | 94 (21) | 95 (24) | 94 (20) | 141 |
| 29 | 96 (19) | 98 (23) | 98 (19) | 116 |
| 30 | 94 (19) | 98 (23) | 95 (19) | 145 |
| 31 | 95 (21) | 97 (22) | 97 (21) | 118 |
| 32 | 95 (20) | 98 (24) | 96 (22) | 143 |
| 33 | 93 (21) | 97 (26) | 96 (21) | 122 |
| 34 | 95 (19) | 97 (23) | 95 (20) | 144 |
| 35 | 93 (20) | 96 (22) | 95 (22) | 122 |
| 36 | 96 (21) | 100 (25) | 97 (21) | 144 |
| 37 | 94 (21) | 96 (24) | 96 (24) | 116 |
| 38 | 94 (18) | 97 (22) | 95 (20) | 146 |
| 39 | 93 (21) | 96 (22) | 95 (22) | 114 |
| 40 | 93 (19) | 95 (24) | 94 (20) | 140 |
| 41 | 92 (20) | 95 (21) | 94 (21) | 125 |
| 42 | 95 (19) | 98 (25) | 96 (21) | 131 |
| 43 | 93 (22) | 96 (26) | 95 (24) | 114 |
| 44 | 94 (18) | 98 (23) | 96 (20) | 135 |
| 45 | 94 (22) | 98 (25) | 97 (22) | 113 |
| 46 | 94 (18) | 97 (22) | 97 (20) | 133 |
| 47 | 97 (23) | 98 (26) | 98 (23) | 104 |
| 48 | 95 (20) | 99 (24) | 96 (21) | 129 |
| 49 | 95 (21) | 98 (25) | 97 (20) | 111 |
| 50 | 96 (22) | 99 (23) | 98 (23) | 125 |
| 51 | 95 (24) | 99 (30) | 97 (25) | 99 |
| 52 | 98 (23) | 102 (27) | 99 (24) | 119 |
| 53 | 98 (22) | 101 (26) | 100 (22) | 96 |
| 54 | 96 (21) | 99 (25) | 97 (23) | 117 |
| 55 | 99 (21) | 101 (24) | 101 (21) | 96 |
| 56 | 95 (20) | 101 (25) | 97 (20) | 102 |
| 57 | 95 (22) | 98 (25) | 98 (22) | 99 |
| 58 | 97 (22) | 99 (25) | 98 (19) | 94 |
| 59 | 98 (23) | 101 (25) | 99 (22) | 95 |
| 60 | 97 (21) | 102 (26) | 99 (21) | 93 |
| 61 | 96 (21) | 100 (25) | 99 (22) | 87 |
| 62 | 97 (21) | 100 (24) | 98 (20) | 98 |
| 63 | 94 (21) | 98 (26) | 97 (23) | 85 |
| 64 | 98 (21) | 101 (24) | 98 (22) | 87 |
| 65 | 98 (21) | 99 (24) | 99 (23) | 75 |
| 66 | 99 (21) | 101 (26) | 100 (21) | 89 |
| 67 | 97 (20) | 98 (26) | 98 (22) | 62 |
| 68 | 97 (23) | 99 (26) | 98 (22) | 91 |
| 69 | 96 (23) | 99 (28) | 98 (24) | 62 |
| 70 | 99 (21) | 99 (23) | 98 (21) | 73 |
| 71 | 96 (21) | 96 (25) | 97 (22) | 63 |
| 72 | 99 (22) | 102 (27) | 101 (23) | 65 |
| 73 | 95 (22) | 99 (27) | 97 (23) | 53 |
| 74 | 96 (22) | 99 (26) | 98 (22) | 47 |
| 75 | 97 (20) | 100 (25) | 98 (21) | 57 |
| 76 | 98 (20) | 100 (26) | 98 (22) | 51 |
| 77 | 93 (20) | 98 (23) | 96 (22) | 47 |
| 78 | 100 (20) | 104 (26) | 101 (21) | 45 |
| 79 | 92 (19) | 96 (22) | 94 (20) | 40 |
| 80 | 100 (19) | 100 (28) | 99 (19) | 39 |
| 81 | 95 (19) | 96 (23) | 97 (21) | 36 |
| 82 | 102 (18) | 103 (23) | 101 (18) | 28 |
| 83 | 96 (21) | 99 (26) | 100 (24) | 35 |
| 84 | 99 (17) | 101 (23) | 100 (17) | 25 |
| 85 | 94 (19) | 96 (25) | 98 (22) | 28 |
| 86 | 100 (18) | 101 (23) | 99 (20) | 23 |
| 87 | 97 (20) | 97 (25) | 97 (24) | 24 |
| 88 | 100 (22) | 93 (24) | 98 (21) | 22 |
| 89 | 94 (19) | 95 (23) | 97 (24) | 21 |
| 90 | 100 (20) | 101 (28) | 99 (22) | 21 |
| 91 | 90 (20) | 91 (18) | 95 (24) | 15 |
| 92 | 98 (17) | 95 (21) | 96 (17) | 18 |
| 93 | 95 (21) | 103 (27) | 100 (26) | 15 |
| 94 | 96 (16) | 100 (23) | 99 (18) | 16 |
| 95 | 93 (20) | 97 (24) | 99 (28) | 11 |
| 96 | 93 (14) | 98 (28) | 95 (20) | 11 |
| 97 | 94 (24) | 104 (19) | 100 (14) | 7 |
| 98 | 96 (29) | 106 (41) | 103 (32) | 10 |
| 99 | 86 (22) | 91 (24) | 86 (20) | 4 |
| 100 | 87 (26) | 90 (37) | 93 (24) | 7 |
| 101 | 95 (16) | 103 (16) | 98 (7) | 3 |
| 102 | 81 (29) | 81 (32) | 81 (33) | 6 |
| 103 | 90 (17) | 103 (16) | 97 (7) | 3 |
| 104 | 82 (25) | 89 (40) | 84 (32) | 4 |
| 105 | 84 (16) | 86 (20) | 84 (18) | 4 |
| 106 | 106 | 111 | 107 | 1 |
| 107 | 78 (20) | 80 (24) | 81 (19) | 5 |
| 108 | 110 | 121 | 116 | 1 |
| 109 | 89 (21) | 90 (22) | 88 (15) | 4 |
| 110 | 94 (7) | 104 (17) | 95 (13) | 2 |
| 111 | 93 (19) | 90 (20) | 86 (21) | 3 |
| 112 | 95 (5) | 100 (20) | 92 (14) | 2 |
| 113 | 82 (4) | 90 (22) | 83 (15) | 2 |
| 114 | 106 | 125 | 107 | 1 |
| 115 | 83 (1) | 99 (36) | 88 (13) | 2 |
| 116 |  |  |  | 0 |
| 117 | 88 (10) | 84 (20) | 85 (17) | 2 |
| 118 |  |  |  | 0 |
| 119 | 83 (14) | 86 (27 | 80 (20) | 2 |
| 120 |  |  |  | 0 |
| 121 | 86 (10) | 88 (30) | 82 (17) | 2 |
| 122 |  |  |  | 0 |
| 123 | 81 (9) | 78 (18) | 74 (15) | 2 |
| 124 |  |  |  | 0 |
| 125 | 88 (8) | 87 (24) | 84 (17) | 2 |
| 126 |  |  |  | 0 |
| 127 | 98 | 109 | 98 | 1 |
| 128 |  |  |  | 0 |
| 129 | 86 | 112 | 94 | 1 |
| 130 |  |  |  | 0 |
| 131 | 93 | 114 | 98 | 1 |
| 132 |  |  |  | 0 |
| 133 | 96 | 106 | 100 | 1 |
| 134 |  |  |  | 0 |
| 135 | 101 | 105 | 99 | 1 |
| 136 |  |  |  | 0 |
| 137 | 96 | 111 | 96 | 1 |
| 138 |  |  |  | 0 |
| 139 | 99 | 117 | 101 | 1 |
| 140 |  |  |  | 0 |
| 141 | 86 | 106 | 90 | 1 |
| 142 |  |  |  | 0 |
| 143 | 90 | 102 | 90 | 1 |
| 144 |  |  |  | 0 |
| 145 | 96 | 103 | 94 | 1 |
| 146 |  |  |  | 0 |
| 147 | 96 | 108 | 98 | 1 |
| 148 |  |  |  | 0 |
| 149 | 83 | 109 | 89 | 1 |
| 150 |  |  |  | 0 |
| 151 | 95 | 114 | 102 | 1 |
| 152 |  |  |  | 0 |
| 153 | 94 | 108 | 96 | 1 |
| 154 |  |  |  | 0 |
| 155 | 93 | 103 | 95 | 1 |
| 156 |  |  |  | 0 |
| 157 | 99 | 97 | 97 | 1 |

**Supplementary table 1 The mean (SD) relative falls in systolic, mean and diastolic blood pressures over time compared to baseline (spinal anaesthesia infiltration at t0 (i.e. the primary objective)). These data are shown graphically in Fig. 2, excluding data points averaged from fewer than 10 patients (shown in red above).**
